# Precarious work and chronic disease: lessons learned from the US national agricultural worker survey (1999–2020)

**DOI:** 10.3389/fpubh.2025.1651197

**Published:** 2025-09-17

**Authors:** Lisbeth Iglesias-Rios, Kexin Li, Alexis J. Handal, Lu Wang

**Affiliations:** ^1^Department of Epidemiology, School of Public Health, University of Michigan, Ann Arbor, MI, United States; ^2^Department of Biostatistics, School of Public Health, University of Michigan, Ann Arbor, MI, United States

**Keywords:** precarious employment, chronic disease, farmworkers, health inequalities, health disparities

## Abstract

**Background:**

Precarious employment and labor exploitation in farmworkers is historical and pervasive in the United States.

**Methods:**

We analyzed cross-sectional data from the National Agricultural Worker Survey (1999–2020, NAWS) among 46,910 farmworkers. A multidimensional precarious employment score (PES) was developed using work indicators and cardiovascular risk factors and cardiovascular disease (CVD). Analysis included: (1) Poisson regression model with LASSO for key predictor selection across three health outcomes; (2) PES construction to track employment conditions over time; and (3) subgroups analyses to assess disparities and trends in employment precarity.

**Results:**

Women showed no reduction in PES over time. Indigenous farmworkers [mean (*M*) = 0.52, 95% CI: 0.50 to 0.53; difference from reference (δ*M*) = 0.07, 95% CI: 0.05 to 0.08] and those working with labor contractors (*M* = 0.5, 95% CI: 0.48 to 0.52; δ*M* = 0.05, 95% CI: 0.04 to 0.07) showed a consistently higher PES, but their declines [Indigenous: change (Δ*M*) = −0.05, 95% CI: −0.09 to 0; labor contractors: Δ*M* = −0.08, 95% CI: −0.12 to −0.05) were smaller.

**Conclusions:**

Within this precarious workforce there were differences in PES and chronic disease. Biosocial data is needed to better understand the pathways of how precarious employment impacts the health of this workforce.

## 1 Introduction

Farmworkers in the United States (US) continued to face precarious and exploitative work conditions despite agriculture, food and its related industries contributing ~$1.5 trillion to the gross domestic product in 2023 (1). There is substantial evidence that workers under precarious work conditions generally face job insecurity, low wages, limited workplace rights, high demands and lower control over their work environment all salient characteristics of farmworker work conditions (2–5) that have been associated with higher levels of chronic stress and adverse health outcomes (e.g., cardiovascular risk factors and cardiovascular disease) compared to workers in more secure or stable work environments (6–16).

Social and economic marginalization are associated with chronic diseases and lower life-expectancy (14, 15, 17, 18) yet, scarce research has advanced the conceptualization and measurement of precarious work and labor exploitation for these workers with chronic disease. This is a needed step to advance health equity and policies to ensure worker safety and health.

Within the literature of precarious work and its measurement, the Western European and North American research on salaried workers with more standard work arrangements prevails (19–21). Yet, work precariousness and exploitative labor for farmworkers entails a different social and complex reality interwoven with the lack or minimal social and labor protections for these workers and the criminalization and contentious immigration policies of the US (22, 23). The most recent report from the only employment-based US survey on farmworkers, the National Agricultural Work Survey (NAWS), in 2021–2022 (24), indicated that most workers are foreign-born mainly from Mexico (~80%), almost two quarters (~40%) are undocumented and very few receive federal contribution-based benefits (disability insurance, unemployment insurance, social security) (12%), despite being in the US approximately for 10 years or more (24). The International Labour Organization (ILO) (25), the US National Institute for Occupational Health (NIOSH) through its Total Worker Health initiative (26, 27) and scholars (6, 7, 21, 28) have all called for the need to define precarious employment holistically, incorporating the built environment (e.g., safe and secure facilities), management practices (e.g., healthy leadership), organization of work (e.g., adequate breaks, stress prevention), and the psychosocial work environment (e.g., autonomy, flexibility, empowerment of workers).

In an attempt to develop such understanding with farmworkers, our previous research with migrant, seasonal and H-2A farmworkers identified the following dimensions of precarious employment and labor exploitation: organization of work, wages, work environment, workplace dynamics, leadership; power and control; social vulnerability (2–5). Building on this work and the existent literature, the present analysis used survey data from the NAWS (1999–2020) to quantify key influential employment characteristics of precarious work and chronic disease. The purpose of this study was twofold: (1) to create a multidimensional precarious employment score (PES) based on its association with the prevalence of chronic disease (diabetes, hypertension and cardiovascular disease) in migrant and seasonal farmworkers, and (2) to describe changes in PES, both overall (e.g., women compared to men) and within subgroups (e.g., within women).

## 2 Methods

We used cross-sectional data from the NAWS, a US Department of Labor survey on work characteristics of crop migrant and seasonal farmworkers. NAWS sampling methodology is described in detail on the US Department of Labor website (29). Briefly, NAWS uses a complex, multistage sampling methodology to capture regional variation across 12 US regions, consolidated into six: California, Southwest, Southeast, Northwest, Midwest and East. NAWS has seven sampling levels: cycle, region, farm labor area (primary sampling unit), county, ZIP code, employer, and farmworker. Regions and farms are randomly sampled based on the amount of farm labor during a given cycle (29).

Our original NAWS dataset included 71,311 individuals from 1988 to 2020. For demographic analysis, we restricted to individuals aged 18 years or older between 1989 and 2020 (*n* = 68,445). To construct the Precarious Employment Score (PES), we further restricted the sample to 46,910 individuals from 1999 to 2020, when cardiovascular relevant indicators for this analysis became available, and included only those with complete demographic data (e.g., gender, age, NAWS sampling cluster). Given the survey's complexity, complete case analysis was conducted to avoid the potential bias of the imputation model.

### 2.1 Exposure: Precarious employment score (PES)

We constructed a PES based on literature and our previous research with farmworkers (2–5). The PES includes five dimensions: material assets, work and living arrangements, job security, worker benefits, and social vulnerability, with 17 variables from NAWS data (Table 1). Each dimension includes socio-demographic and employment variables, scored for precariousness. No existing measure of precarious work for farmworkers has been documented, and previous research mainly focused on urban workers with standardized work conditions (19, 20, 28). Given our interest in chronic disease, we based the PES on self-reported cardiovascular risk factors (diabetes, hypertension) and cardiovascular disease (CVD), given the strong evidence that precarious work contributes to CVD through various pathways, and NAWS data consistently collected these health outcomes from 1999 to 2020 (8, 9, 11, 30–32).

**Table 1:** Dimensions and indicators of precarious employment for farmworkers: data from the National Agricultural Worker Survey (NAWS 1989–2020).

**Dimensions**	**Item**	**Scoring rubric**
(1) Material assets	Below minimum wage	Likely precarious if below minimum wage
	Payment method	Likely precarious if paid by piece-rate or a combination of hourly wage and piece-rate; not precarious if paid by the hour, salary, or other methods
	Family poverty	Likely precarious if family income is below the federal poverty level
	Vehicle ownership^a^	Likely precarious if no vehicle is owned, limited transportation and mobility
(2) Work and living arrangements	Employment status	Likely precarious if employed by a farm labor grower at the time of the interview; not precarious if employed by a contractor
	Dwelling ownership^b^	Likely precarious if no dwelling is owned
	Hours worked last week	Likely precarious if total hours worked per week at current farm job are <20 or >40; not precarious otherwise
(3) Job security-stability	Weeks at current job	Likely precarious if employed at the current job for <6 weeks
	Farmwork days in the previous 12 months	Likely precarious if the number of farmwork days is <= 183 days (6 months)
	Farmwork weeks last year	Likely precarious if <48 weeks of farmwork were completed last year
(4) Worker's benefits (health insurance)	Contribution-based government benefits^c^	Likely precarious if contribution-based government benefits were not received
	Need-based government benefits^d^	Likely precarious if need-based were not received
	Employer healthcare for work-related injuries	Likely precarious if no health insurance is provided
(5) Social vulnerability	Indigenous status	Likely precarious if identified as indigenous
	Place of birth	Likely precarious if not born in the U.S. born
	Current legal status	Likely precarious if worker is undocumented
	Adult primary language	Likely precarious if primary language is anything other than English

a Owning a vehicle in rural areas is key for transportation and access to services.

b Farmworkers who do not own their dwelling typically live in farm headquarters or in rental housing. Lack of dwelling ownership was more prevalent among individuals below the poverty line (38.8% vs. 51.5%) and among those without legal work authorization (43.2% vs. 50.9%), reflecting increased housing vulnerability in these populations.

c Contribution-based government benefits, as defined by NAWS, include disability insurance, unemployment insurance, Social Security, and veterans' pay. Receipt was lower among individuals below the poverty line (13.6% vs. 23.5%) and significantly lower among those without legal work authorization (2.5% vs. 36.9%), supporting its inclusion as an indicator of precariousness.

d Needs-based government benefits include Temporary Assistance for Needy Families (TANF), general assistance or welfare, publicly provided housing, and medical or nutritional programs such as Medicaid, the Special Supplemental Nutrition Program for Women, Infants, and Children (WIC), and the Supplemental Nutrition Assistance Program (SNAP). Receipt was only marginally higher among individuals below the poverty line (43.6% vs. 37.6%) and lower among those without legal work authorization (38.6% vs. 40.3%), indicating limited access among potentially eligible groups.

### 2.2 Outcomes: self-reported cardiovascular risk factors and cardiovascular disease

We focused on three self-reported binary cardiovascular risk outcomes (diabetes, hypertension and cardiovascular disease (CVD). These health outcomes were measured in NAWS from 1999 to 2020 and were assessed as: “Have you ever been told by a doctor or nurse (health practitioner) that you have the following [condition]? Yes/No.”

### 2.3 Statistical analysis

All analyses accounted for the NAWS complex sampling design and weights. We summarized sociodemographic characteristics and estimated the prevalence of self-reported CVD risk factors across key demographic groups, assessing group differences using 95% confidence intervals (CI) and *p*-values. Precarious work variables were dichotomized (0 = not precarious, 1 = precarious). Cardiovascular health outcomes were measured as counts based on data from 1999–2020 (e.g., 0 = none, 1 = 1 condition, 2 = 2 condition, 3 = 3 condition) and modeled using Poisson regression to evaluate their risk factors. A Poisson regression with weighted Least Absolute Shrinkage and Selection Operator (LASSO) regularization was applied to select the most influential variables, addressing multicollinearity (33, 34). Final models were adjusted for well-known confounders, including age, gender, region, farmworker type (migrant, seasonal, or H-2A), ethnicity, education, marital status, family poverty, and health insurance access.

Selected variables were summed with equal weights to construct the PES, following prior studies that applied equal weighting in the absence of a predefined rationale for differential weighting (20, 28). The PES was rescaled from 0 (least precarious) to 1 (most precarious) and adjusted to age 30 to account for aging effects, a period linked to shifts in employment and increased health risks for farmworkers.

We examined changes in PES over time between (e.g., women vs. male) and within (e.g., changes within women) subgroups. The average PES (denoted as M) for each time period (TP) and subgroup. The difference in PES between subgroups at a given time point is denoted by δ*M*, while Δ*M* captures changes in within subgroups over time. We also analyzed the interaction between time and subgroups [δ(Δ*M*)]. Percentage changes in PES from 1999 to 2020 was computed as (PES_TP5_ – PES_TP1_)/PES_TP1_ × 100%.

## 3 Results

### 3.1 Sociodemographic characteristics

Supplementary Table 1 summarizes the sociodemographic characteristics of the cohort. The farmworker population has declined and aged over time (mean age: 33 in 1989, 41 in 2020). California consistently had the highest concentrations of farmworkers with almost half. While the workforce remains predominantly comprised of males (75%) and Latinos(as) (80%); in the past 10 years, we see a steady increase in the proportion of women workers. Indigenous farmworkers have consistently made up approximately 7% of the farmworker population.

Most farmworkers have consistently been Spanish-speaking and foreign born, primarily from Mexico. In 2019–2020, the survey added categories for bilingual Spanish/English speakers (7%) and multilingual individuals (5%). Nearly half (44%) of farmworkers were undocumented, while 30% were US citizens and 27% had work authorization.

Over two-thirds were married, and 65% had minor children, with many having 1–2 (34%), 3 (21%), or 4+ children (11%). About 12% of workers had mixed-status families, where the worker was undocumented but the children were US citizens. Education attainment remained low, with most completing only elementary education.

Poverty remained high among farmworkers, with over two-thirds living below 200% of the federal poverty threshold. Average annual personal income was $14,961, and family income averaged $19,866. Migrant farmworkers consistently represented a smaller proportion, declining sharply since 2011 to 15% by 2020. Approximately 20% were employed by farm labor contractors, consistently fewer than those employed by growers. Supplementary Table 2 presents self-reported cardiovascular risk factors and CVD across key demographic groups. These conditions were more prevalent in older farmworkers (~49 years old) and varied by gender.

### 3.2 Construction of precarious employment score (PES)

Nine precarious work indicators were selected based on LASSO and combined using equal weights (Supplementary Table 3). These indicators comprehensively represented the five original dimensions, which ensured that the constructed PES captures changes across all dimensions of precarious work.

### 3.3 Time trends for precarious employment score (PES) and diabetes, hypertension and cardiovascular disease

**Figure 1:**
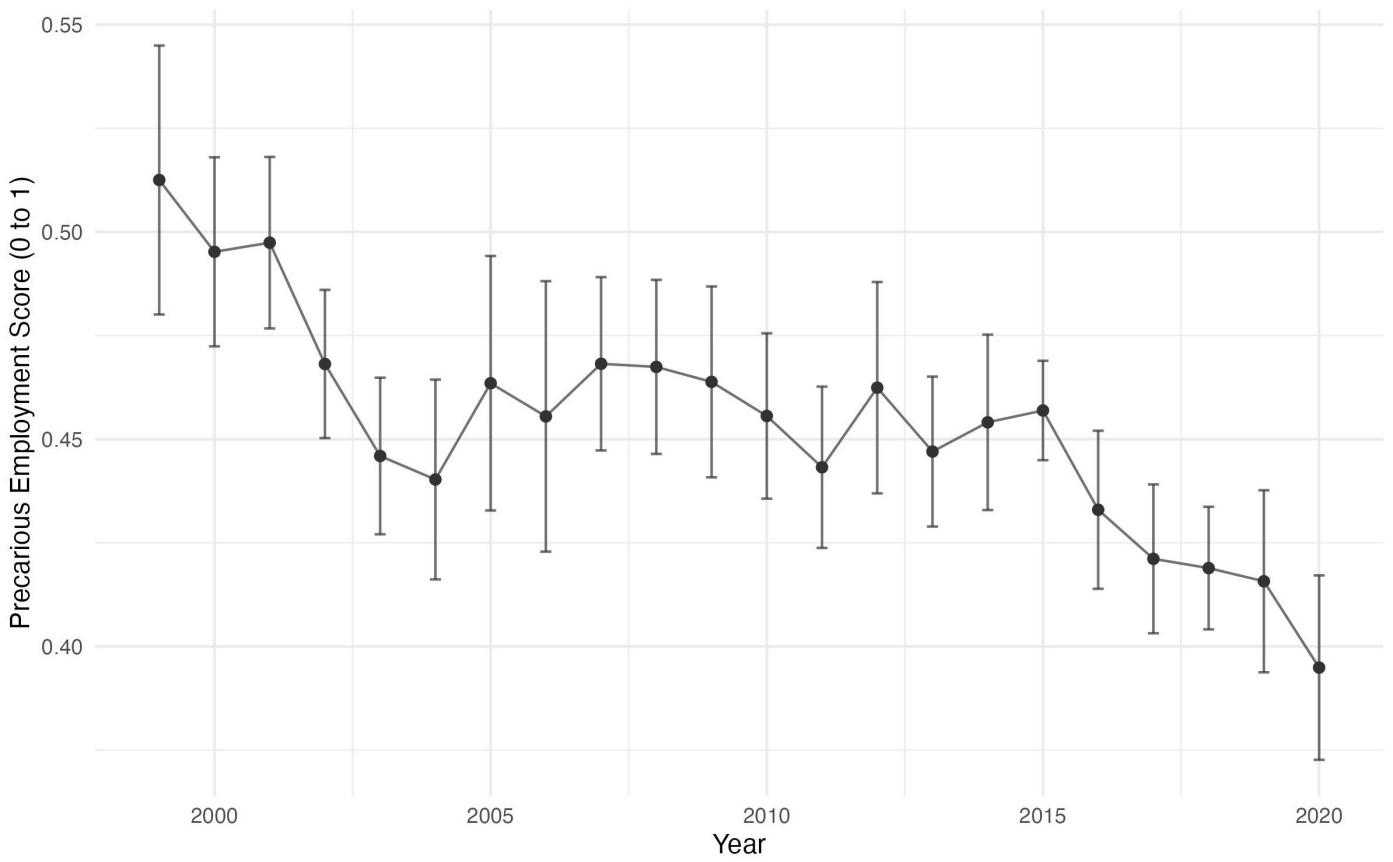
Precarious Employment Score (PES) Over Time from the National Agricultural Worker Survey (NAWS), 1999–2020.

The age-adjusted PES decreased significantly from 1999 to 2002 (TP1) (*M* = 0.49, 95% CI: 0.48 to 0.51) to 2015–2020 (TP5) (*M* = 0.43, 95% CI: 0.42 to 0.43), a 14.3% reduction (Δ*M* = −0.07, 95% CI: −0.09 to −0.05) (Supplementary Table 4) with a fluctuating downward trend (Figure 1).

Gender differences were observed: males had a slightly higher average PES (*M* = 0.46) than females (*M* = 0.44) but experienced a greater decline from 1999–2002 to 2015–2020 (−17.6%, Δ*M* = −0.09, 95% CI: −0.11 to −0.07) compared to no change among females (Δ*M* = 0.0, 95% CI: −0.03 to 0.02). This difference in decline was statistically significant [δ(Δ*M*) = 0.08, 95% CI: 0.05 to 0.12].

Among the six NAWS regions, the East (*M* = 0.47), California (*M* = 0.46), the Southeast (*M* = 0.46), and the Northwest (*M* = 0.46) had similar higher adjusted PES. From TP1 to TP5, the Southwest (−21.6%, Δ*M* = −0.11, 95% CI: −0.14 to −0.07) and Southeast (−17.6%, Δ*M* = −0.09, 95% CI: −0.14 to −0.03) experienced the largest declines.

Subgroup trends showed that foreign-born workers, Latinos, individuals with lower education, and migrant workers had initially higher PES, which declined significantly over time—more than in other groups—yet their overall levels remained higher. For example, migrant farmworkers had a 28% significantly higher PES than seasonal farmworkers (*M* = 0.55 vs. *M* = 0.43; δ*M* = 0.12, 95% CI 0.11 to 0.14). Further, within migrant we observed a higher average PES from TP1 to TP5 and a significantly greater decline in total PES (−15.5%, Δ*M* = −0.09, 95% CI −0.11 to −0.07), with statistically significant difference in the magnitude of the decline [δ(Δ*M*) = −0.07, 95% CI −0.10 to −0.04] when compared to seasonal farmworkers.

However, this trend was not observed among certain groups. Indigenous workers had persistently higher PES than non-Indigenous workers (*M* = 0.52 vs. *M* = 0.45; δ*M* = 0.07, 95% CI: 0.05 to 0.08), with only a slight decline (−9.4%, Δ*M* = −0.05, 95% CI: −0.09 to 0) over the years that was smaller than the decrease for non-Indigenous workers [δ(Δ*M*) = 0.02, 95% CI: −0.02 to 0.07], indicating limited improvements for Indigenous. Similarly, farmworkers employed by farm labor contractors consistently faced poorer employment conditions than those working for growers (*M* = 0.50 vs. *M* = 0.45). Despite a 15% decline (Δ*M* = −0.08, 95% CI: −0.12 to −0.05), the gap remained [δ(Δ*M*) = −0.02, 95% CI: −0.06 to 0.02], reflecting only modest improvements.

### 3.4 Sensitivity analyses

We conducted sensitivity analyses to assess the robustness of our findings. First, we checked if the selected precarious work variables matched our predefined dimensions. Second, we tested age adjustment by using survey wave fixed effects instead of categorical year indicators. Finally, we restricted the sample to individuals with health insurance and those who had received health care services in the US within the past 2 years.

Results confirmed the stability of the PES. The nine selected variables aligned with predefined dimensions, validating the data-driven approach (Supplementary Table 3). Using wave fixed effects produced nearly identical results (Supplementary Table 5). Restricting the sample to individuals with health insurance or healthcare access produced lower PES and slower decline, but the downward trend remained.

## 4 Discussion

To our knowledge, this is the first study that uses employment-based survey data on migrant and seasonal farmworkers to develop a multidimensional PES to further knowledge on the potential impact of precarious work on cardiovascular risk factors and disease.

While prospective data is needed, our findings, consistent with previous studies with non-farmworker populations (9, 12, 14, 16), suggest that precarious work and the clustered forms of marginalization experienced by farmworkers (e.g., high rates of poverty, food insecurity, lack of access to health care) may accelerate health declines due to chronic stress and further the susceptibility of these workers to chronic disease and premature mortality.

In contrast to previous studies assigning arbitrary weights on precarious employment indicators, our methodological approach was guided by our estimates on cardiovascular risk factors and CVD given the strong evidence that these health outcomes are associated with poor work conditions in the general population and this workforce.

Our results are consistent with previous literature and show that over the past 21 years, farmworkers have remained overrepresented by Latinos born outside the US, and continue to be an impoverished population with a strikingly higher number of workers living below the 200% poverty federal level despite having underage children. We found that this workforce is aging, with a difference of a little more than 10 years of age from 1989 to 2020. The aging of this workforce is a concern for the future of agriculture and farming in the US and deserves consideration.

Our analysis showed that almost half of farmworkers were undocumented and while over the years there has been a consistently higher proportion of seasonal farmworkers compared to migrant farmworkers, a steep decline in the number of migrant workers started to occur in 2011 and has continued into 2020. This decline may be explained by the increase in immigration enforcement, deportation raids and heightened fear within immigrant communities accentuated in 2010, impacting the ability for migrant farmworkers to travel for work state-to-state (22, 23, 35). Our prevalence findings across groups are consistent with data from the Behavioral Risk Factor Surveillance System on self-reported diagnosed adjusted prevalence of diabetes being 8.8% (95% CI: 5.9–13.0) for agricultural workers (36). Furthermore, the prevalence of diabetes and high blood pressure reported in our study is consistent with other studies using self-reported health measures for farmworkers, which have reported a prevalence of 8.7–13.5% for diabetes (8, 37) and 12.7% for high blood pressure (8). However, our results are inconsistent with a recent study that employed data from three research studies conducted in California (2009–2017) with anthropometric measures, which showed a prevalence almost 5 times higher for high blood pressure (42 to 45.5%) (30). The inconsistencies in disease prevalence across studies suggest that self-reported health data underestimate prevalence and assessing clinical measures as part of national surveys monitoring the health and safety of farmworkers is warranted.

Our time trend analysis showed that our PES score was associated with chronic disease (diabetes, hypertension and CVD) for key sociodemographic and employment characteristics and only a 14% decreased in PES was observed from 1999 to 2020. Overall, our results showed that that migrant farmworkers, non-US born farmworkers, Latinos and individuals with lower educational attainment have the largest decline in PES over time, but have a persistent higher PES when compared to their counterparts. Little to no improvements were observed generally for women, Indigenous farmworkers, and those who work with farm labor contractors. Indigenous farmworkers and those who work with contractors showed little decline in PES for chronic disease and a persistently worse PES compared to their counterparts.

Our study highlights that the situation for women has not changed over the years, a concerning finding given than women represent a third of the farmworker population in the US. Gendered occupational exposures and social roles can shape health outcomes; however, there are no consistent findings on chronic disease by gender with farmworkers (38, 39). A study using data from three studies in California (PASOS pilot, MICASA, PASOS-RCT) with farmworkers found that women had higher odds of having elevated waist circumference than males (18). Waist circumference is associated with chronic conditions like diabetes, hypertension and with all-cause cardiovascular mortality (18, 40). Several factors related to gender-employment health inequities (e.g., wages, working hours and tasks, behavioral factors) may contribute to the observed differences by gender and chronic disease, but more research is needed in this area.

Regionally, all 6 NAWS regions had similar higher PES for chronic disease with the East showing the higher PES. California, the Southeast, the Midwest, the Southwest, and the Northwest showed a small but statistically significant decline in PES over time, with relatively larger decreases observed in California, the Southwest, and the Southeast. While our results showed that there has been a greater reduction in migrant farmworkers in these three regions, as well as a notable decline in labor contractors in California, regional differences may be attributed by a myriad of state-related policies on farmworkers safety and health. For instance, following the January 2014 Affordable Care Act's (ACA), six states (California, Colorado, Illinois, New York, Oregon, and Washington) have expanded state-funded coverage to some income-eligible adults regardless of immigration status, illustrating the varying levels of access to health care services across states for farmworkers (41). Overall, our results showed that while some changes in farmworkers employment conditions and health have occurred over the past 21 years, there are striking PES differences within this population. The double burden of high PES and chronic disease for farmworkers is an important health equity gap that requires comprehensive policies and regulatory actions to support labor and social rights for farmworkers.

### 4.1 Strengths and limitations

The study strengths included the use of LASSO, a data-driven approach that presents a relatively novel application in this topic, to derive a multidimensional PES for farmworkers based on cardiovascular risk factors and CVD. To our knowledge, this is the first study that attempts to quantify PES with a large sample size and with 21 years of data from NAWS. The main limitations of the study are the cross-sectional nature of the data and the self-reported health measures that may underestimate the true disease prevalence. However, sensitivity assessments showed consistency in our results. Selection bias is an inherent limitation in NAWS. For example, in years 2019–2022, 52% of the randomly selected eligible growers agreed to participated with the survey and most of the workers sampled (92%) agreed to be interviewed (personal communication NAWS, Sept 30,2024). It is possible that farmworkers from farms where growers declined participation may have worse work conditions and a higher prevalence of disease, thus underestimating our reported findings.

## 5 Conclusion

Current labor policies and occupational health standards for farmworkers must address their complex social, economic, and political realities. The study shows that precarious work is a key pathway for health inequities and even within this population of disadvantaged workers, may not be the same for all workers. The results highlight the need for further research on precarious work and chronic disease integrating biosocial data on farmworkers' employment and health to support the NIOSH Total Worker Health initiative of holistically promoting safety and well-being in all workers.

## Data Availability

Publicly available datasets were analyzed in this study with authorization from NAWS. This data can be found here: https://www.dol.gov/agencies/eta/national-agricultural-workers-survey.
